# Potential causal association between aspirin use and erectile dysfunction in European population: a Mendelian randomization study

**DOI:** 10.3389/fendo.2023.1329847

**Published:** 2024-01-08

**Authors:** Rongkang Li, Lei Peng, Dashi Deng, Guangzhi Li, Song Wu

**Affiliations:** ^1^ Institute of Urology, Lanzhou University Second Hospital, Lanzhou University, Lanzhou, China; ^2^ Institute of Urology, The Affiliated Luohu Hospital of Shenzhen University, Shenzhen University, Shenzhen, China; ^3^ Institute of Urology, South China Hospital, Health Science Center, Shenzhen University, Shenzhen, China

**Keywords:** Mendelian randomization, aspirin, erectile dysfunction, GWAS, causal analysis

## Abstract

**Background:**

Aspirin, as one of the most commonly used drugs, possesses a broad spectrum of therapeutic applications. Presently, the potential association between aspirin usage and the risk elevation of erectile dysfunction (ED) remains inconclusive. The objective of this study employing two-sample Mendelian randomization (MR) was to clarify the causal impact of aspirin use on the risk of ED.

**Methods:**

This study incorporated two sets of Genome-Wide Association Study (GWAS) summary statistics, one for aspirin use (46,946 cases and 286,635 controls) and another for ED (6,175 cases and 217,630 controls) in individuals of European ancestry. The inverse-variance weighted (IVW) method was employed as the primary approach, supplemented by MR-Egger, weighted median, weighted mode, and simple mode to estimate the causal effect of aspirin usage on the risk of ED development. To assess pleiotropy, the MR-PRESSO global test and MR-Egger regression were used. Cochran’s Q test was adopted to check heterogeneity, and the leave-one-out analysis was performed to confirm the robustness and reliability of the results.

**Results:**

The causal association between genetically inferred aspirin use and ED was found by using inverse variance weighted (OR = 20.896, 95% confidence interval = 2.077-2.102E+2, P = 0.010). The sensitivity analysis showed that no pleiotropy and heterogeneity was observed. Furthermore, the leave-one-out analysis demonstrated that the findings were not significantly affected by any instrumental variables.

**Conclusion:**

The results of this study highlighted the significance of aspirin use as a predisposing factor for ED and provided further evidence supporting the causal association between aspirin utilization and ED within European populations.

## 1 Introduction

Erectile dysfunction (ED), a highly prevalent disorder among men, is characterized by the persistent inability to achieve or maintain a penile erection, commonly inducing enormous stress within their intimate relationship (1, 2). ED predominantly, affects males aged 40-70 (~52%) and experiences varying degrees of symptom (3, 4). In the United States alone, it is estimated that at least 12 million males within the age range of 40 to 79 are afflicted by ED (5). This condition poses a significant burden on males, transforming into a health concern that necessitates prompt attention. Consequently, the identification of risk elements associated with ED and the evaluation of individuals who may benefit from proactive prevention or early intervention become imperative.

Aspirin, with a 100-year application history, is one of the most universally employed medications due to its extensive array of therapeutic applications in arthritis, joint discomfort, muscle pain, long-term musculoskeletal pain, febrile conditions, acute or chronic pain, and dysmenorrhea (6, 7). Furthermore, aspirin is recognized as a vital prophylactic agent for CVD and atherosclerotic diseases, and its potential benefits against stroke, thrombosis, and cancer progression are being studied (8, 9). Currently, Aspirin has emerged as a prominent self-administered medicine with widespread usage among patients across different age groups. Nevertheless, the potential influences of aspirin on erectile function also become a subject of contention. Aspirin directly enhances the functionality of endothelial NO synthase, leading to increased production of NO and subsequent smooth muscle relaxation, thereby enhancing vascular blood flow (10). On the other hand, aspirin inhibits the cyclooxygenase (COX) pathway, resulting in reduced levels of vasodilatory agents such as prostaglandin I2 (PGI2) and prostaglandin E2(PGE2) (8), it is plausible to supposed that aspirin may be adversely affect normal erectile function (11). Clinical studies have also yielded different results on this issue (6, 12, 13). Hence, the association between aspirin usage and the risk of ED remains inconclusive.

Mendelian randomization (MR) analysis utilizes the random distribution of parental alleles to offspring during conception. This methodology employs instrumental variables(IVs) to ascertain associations between genotype, intermediate phenotype, and disease outcome, thus enabling the inference of causal relationships between exposure and outcome (14). Similar to randomized controlled trials (RCT), the stochastic allocation of alleles is akin to random sample grouping in RCT and has the potential to address confounding factors that may distort the results (15). MR, utilizing genetic variations as IVs, offers a natural RCT, expanding the epidemiological research methods. The increasing dimensions and range of genome-wide association studies (GWAS) have facilitated the widespread application of two-sample MR analyses across a wide range of diseases (16, 17). In this study, we conducted a two-sample MR analysis utilizing summary statistics derived from large-scale GWAS to assess the causal relationship between aspirin use and ED.

## 2 Methods

### 2.1 Study design and ethics statement

**Figure 1:**
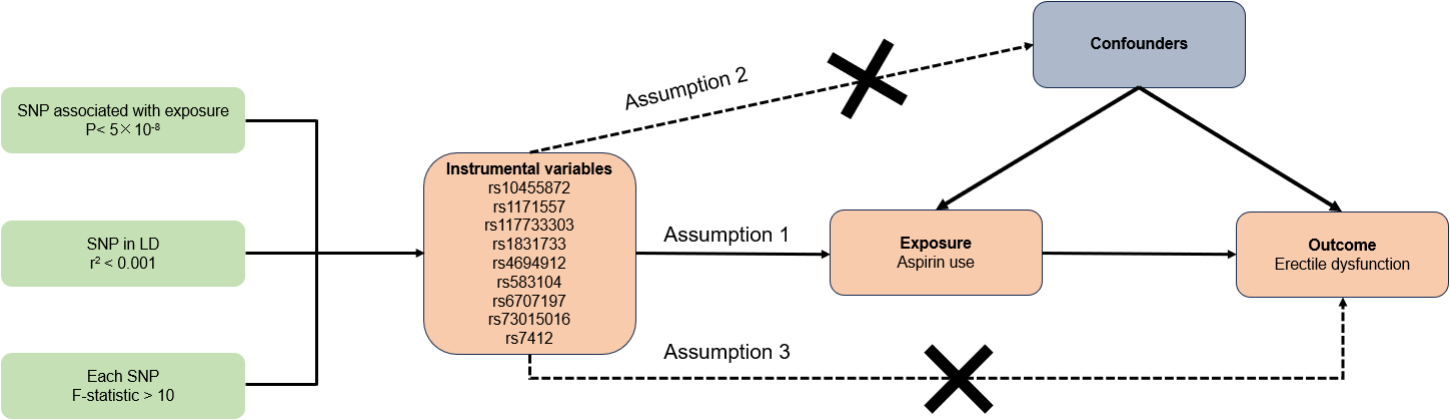
Overall design of this MR analyses.

The overview of our study design and three essential assumptions of the MR study were depicted in **Figure 1**. Assumptions 1 stated that IVs bear a reliable correlation with aspirin use. Assumptions 2 posits that IVs should not be impacted by any confounders. Assumptions 3 maintains that IVs influence the risk of ED solely via aspirin use and not through alternative pathways (18). Given that our data were sourced from existing studies or publicly accessible databases, it was not necessary to seek further ethical clearance from an institutional review board.

### 2.2 Data source

In our research, we designated aspirin use as the exposure and ED as the outcome. Genetic variants linked to aspirin use were identified using the UK Biobank’s GWAS study of aspirin use (ukb-a-452) with a sample size of 333,581. To circumvent population overlap in exposure and outcome, we utilized summary data from an openly available GWAS study of ED (ebi-a-GCST006956) with a sample size of 223,805 (19). ED was defined as self-reported or physician-reported ED using ICD10 codes N48.4 and F52.2, or use of oral ED medication (sildenafil/Viagra, tadalafil/Cialis, or vardenafil/Levitra), or a history of surgical intervention for ED (using OPCS-4 codes L97.1 and N32.6) (19). Both GWAS subjects are individuals with European ancestry. The two GWAS summary data were provided by the IEU open GWAS database and the detailed information were shown in **Table 1**.

**Table 1:** Characteristics of Aspirin use and Erectile dysfunction GWAS cohorts.

Exposure/Outcome	IEU GWAS id	Cases	Controls	Sample size	Number of SNPs	First Author	Population	Year
Aspirin use	ukb-a-452	46,946	286,635	333,581	10,894,596	Neale	European	2017
Erectile dysfunction	ebi-a-GCST006956	6,175	217,630	223,805	9,310,196	Bovijn J	European	2018

### 2.3 Selection of instrumental variables

Relying on the aforementioned GWAS summary data, a stringent procedure was employed to identify suitable single nucleotide polymorphisms (SNPs) as IVs. Initially, SNPs needed to exhibit a strong correlation with exposure at a genome-wide significance level of P-value < 5×10^-8^. Subsequently, to mitigate biased outcomes induced by linkage disequilibrium (LD), a clumping process was carried out with an r^2 ^= 0.001 cutoff and a window size of 10,000 kb. The Phenoscanner database (version 2.0) (http://www.phenoscanner.medschl.cam.ac.uk/) was then utilized to sieve genetic variants associated with confounding factors, including diabetes, obesity, education, sleeplessness or insomnia, psychiatric factors such as anxiety, depression, and bipolar disorder, and removing SNPs associated with any of these potential confounders on a genome-wide basis (20–24). If the previously screened SNPs were not present in the outcome GWAS data, proxy SNPs (high LD of r^2^ >0.8 with the target SNPs) would be sought as replacements. Lastly, to constrain palindromic and ambiguous SNPs with nonconcordant alleles, the exposure and outcome datasets were harmonized, thereby maintaining uniformity of effect alleles on the exposure and outcome. Moreover, to circumvent bias from weak IVs, we computed the F-statistic with the equation F = β^2^ exposure/SE^2^ exposure (25). If the IVs’ F-statistic considerably exceeds 10, the likelihood of bias from weak IVs is minimal (26, 27).

### 2.4 Statistical analysis

In our research, multiple methods were utilized to ascertain and quantify the causal relationships and effects between exposure and outcome. These included the inverse-variance weighted (IVW) (28), MR-Egger (29), simple mode (30), weighted mode (31), and weighted median (32). The IVW method, frequently employed in MR analysis, including random-effects and fixed-effects versions. Operating as a meta-analysis method, IVW combines Wald estimates of causality for each IV to derive overall effect estimates of exposure on outcome. In scenarios devoid of heterogeneity and pleiotropy, the IVW results are deemed most reliable (33). In cases of significant heterogeneity among IVs (P < 0.05), a random effect model was adopted, whereas a fixed effect model was used otherwise. The MR-Egger regression approach delivers trustworthy estimates when pleiotropy is present among IVs (34). The weighted median method continues to generate causal estimates even when less than 50% of the IVs violate key MR assumptions (32). When the majority of IVs are valid, the weighted model approach can conduct MR causal inference (31). The simple mode is less robust than IVW. The aforementioned analyses were conducted and visualized using R software (version 4.3.1), with relevant R packages including “MRPRESSO” (version 1.0) and “TwoSampleMR” (version 0.5.7). A P-value < 0.05 was deemed significant.

### 2.5 Sensitivity analysis

We employed the MR-PRESSO global test and MR-Egger regression to assess the pleiotropy of IVs, considering them to exhibit pleiotropy when P < 0.05 (29, 35). Heterogeneity was measured using Cochran’s Q statistic and considered significant when P < 0.05. Furthermore, we conducted the ‘leave-one-out’ sensitivity analysis to determine if any individual SNP could introduce bias affecting the overall causal effect.

## 3 Results

### 3.1 Selection of instrumental variables

According to the strict evaluation standards for instrumental SNPs, we identified 9 SNPs (rs10455872, rs1171557, rs117733303, rs1831733, rs4694912, rs583104, rs6707197, rs73015016, rs7412) that exhibited a strong association with aspirin usage. These SNPs served as IVs for aspirin use, and each SNP demonstrated an F-statistic exceeding 10, suggesting a minimal risk of weak IV bias. Detailed information of included SNPs was showed in **Supplementary Table 1**.

### 3.2 Mendelian randomization results

**Figure 2:**
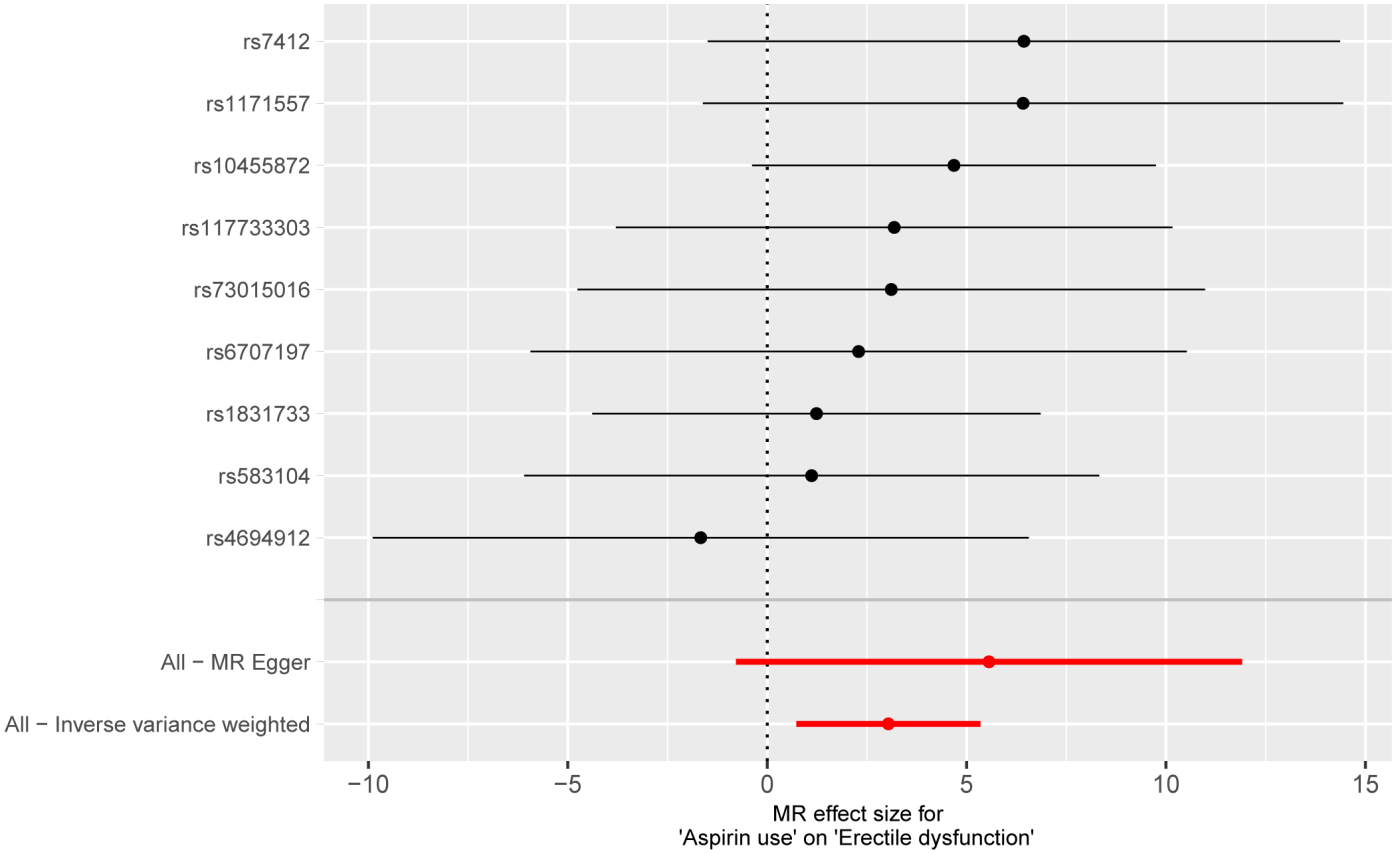
Forest plot for the causality of each SNP on erectile dysfunction risk.

**Figure 3:**
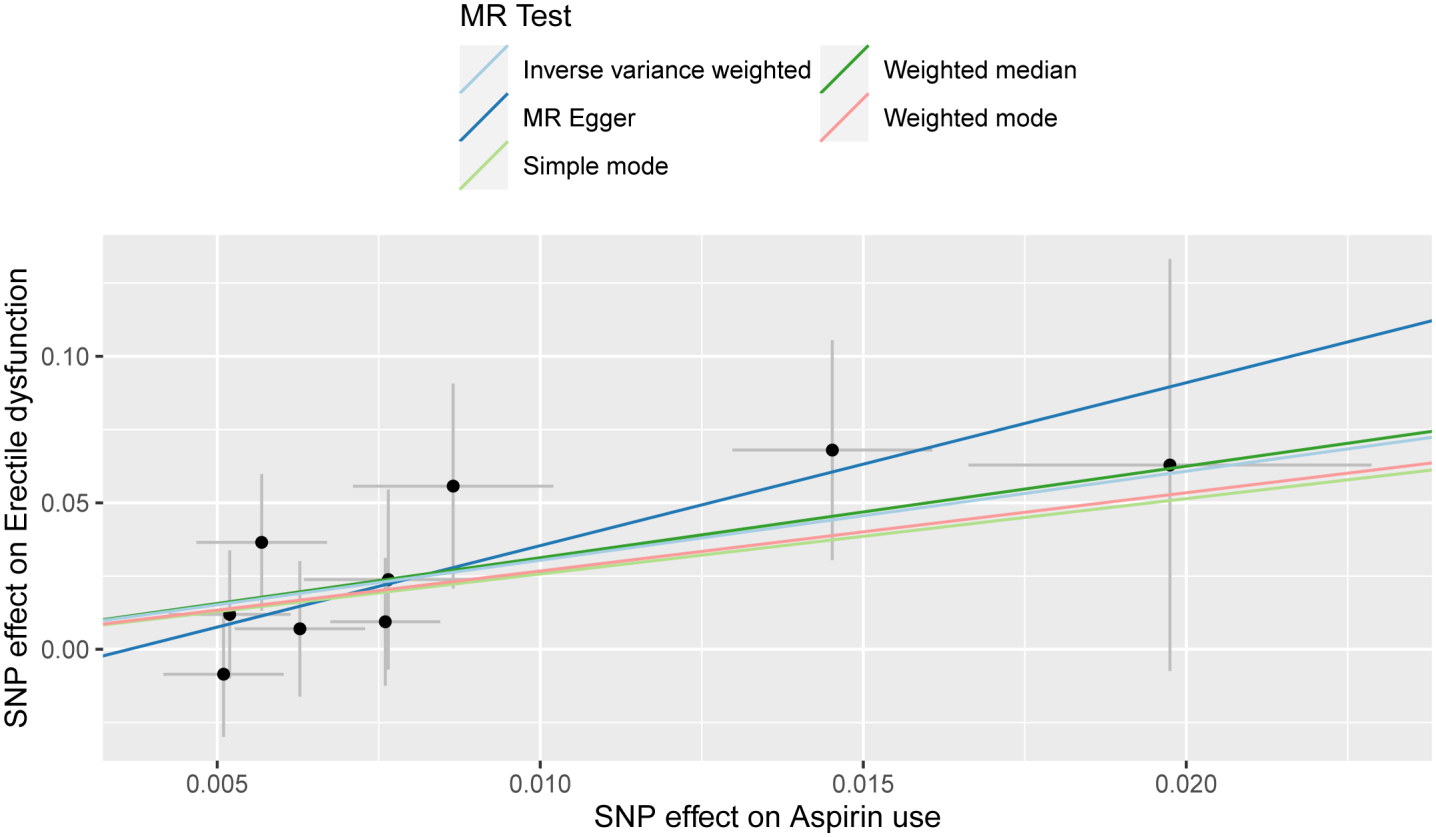
Scatter plot for the causality of aspirin use on erectile dysfunction risk. The regression slopes of the lines represent the magnitude of the causal effect.

The outcomes of the Mendelian randomization analysis were summarized in **Table 2**. IVW revealed a statistical significance of causal impact of aspirin use on the risk of ED (OR = 20.896, 95% confidence interval (CI) = 2.077-2.102E+2, P = 0.010). A similar trend was observed through the weighted median method (OR = 22.768, 95% CI = 1.184-4.378E+2, P = 0.038). These results were visually presented in the forest plot (**Figure 2**) and scatter plot (**Figure 3**). The forest plot displayed the effect estimates and their confidence intervals for each SNP, while the scatter diagram depicted the association between the exposure (aspirin use) and the outcome (ED) utilizing the instrumental variables.

**Table 2:** MR analysis of the causality of Aspirin use on Erectile dysfunction.

Exposure	Outcome	MR method	Number of SNPs	β	SE	OR (95% CI)	P‐value
Aspirin use	ED	MR Egger	9	5.562	3.237	260.37(0.457-1.483E+5)	0.129
Aspirin use	ED	Weighted median	9	3.125	1.508	22.768(1.184-4.378E+2)	0.038
Aspirin use	ED	Inverse variance weighted	9	3.040	1.178	20.896(2.077-2.102E+2)	0.010
Aspirin use	ED	Simple mode	9	2.571	2.358	13.076(0.128-1.331E+3)	0.307
Aspirin use	ED	Weighted mode	9	2.672	2.199	14.469(0.194-1.078E+3)	0.259

ED, Erectile dysfunction; SNPs, single-nucleotide polymorphisms; SE, standard error; OR, odds ratio; CI, confidence interval.

### 3.3 Sensitivity analysis

**Figure 4:**
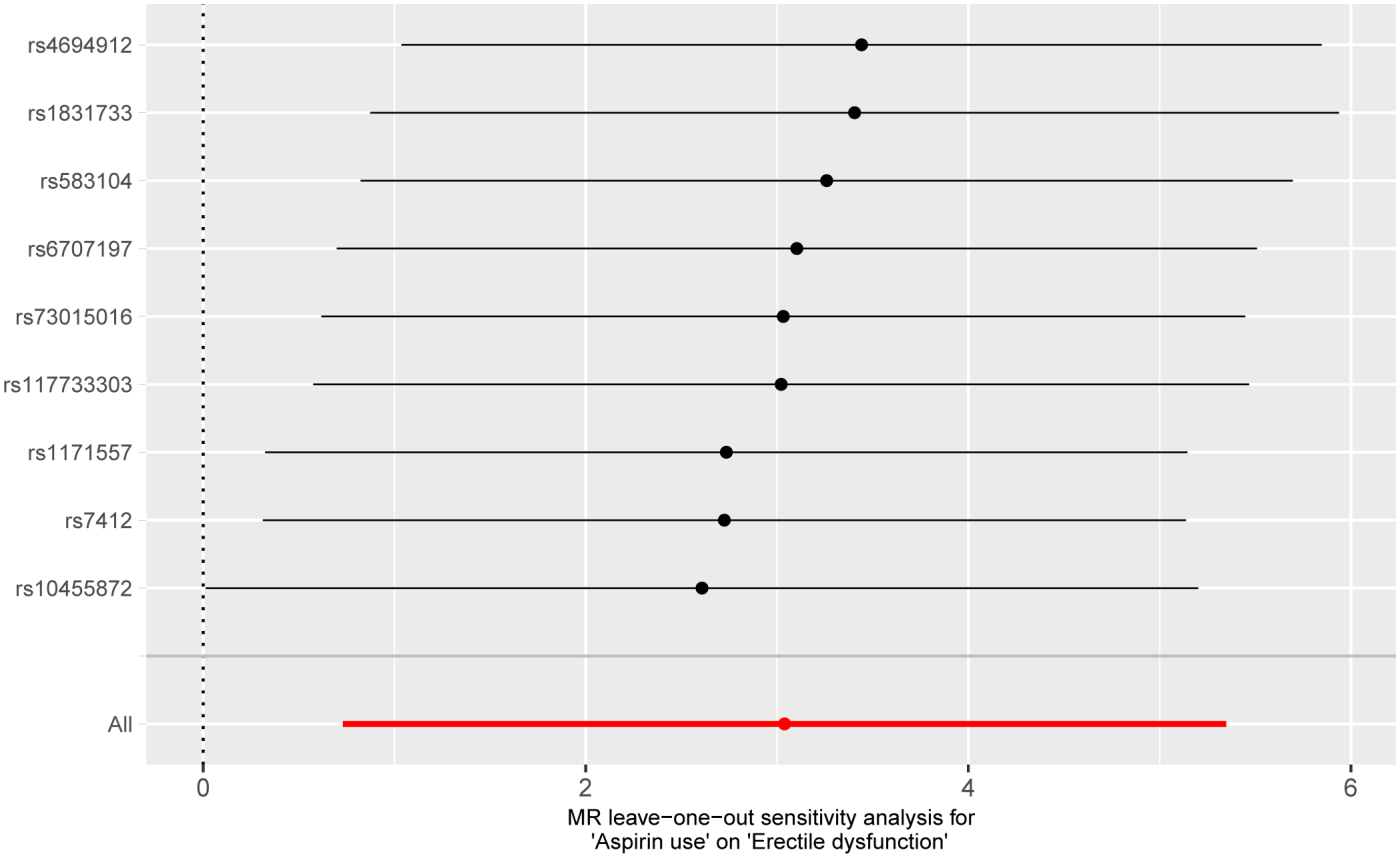
Forest plot for leave-one-out analysis of the effect of aspirin use on erectile dysfunction.

Pleiotropy of IVs was examined through the application of MR-Egger regression and MR-PRESSO global test. The MR-Egger regression inferred that no pleiotropy in IVs (P = 0.430, **Table 3**), a finding that was further supported by the MR-PRESSO global test (P = 0.892, **Table 3**). Cochran’s Q test was employed to identify heterogeneity in IVs. The analysis revealed no heterogeneity based on the Cochran Q-test (P = 0.880 for MR-Egger; P = 0.879 for IVW) (**Table 3**) and funnel plots (**Supplementary Figure 1**). A leave-one-out analysis was conducted, sequentially excluding each SNP to assess its effect on the results. This analysis indicated that none of the individual SNP had a significant impact on the causal inference (**Figure 4**), suggesting that the observed association between aspirin use and ED was not driven by any specific SNP. This highlights the strength and reliability of the results, instilling confidence in the validity and robustness of the causal inference derived from the MR analysis.

**Table 3:** Sensitivity analyses of MR.

Pleiotropy				Heterogeneity			
MR-PRESSO global outlier test		MR-Egger regression		MR-Egger		Inverse variance weighted (IVW)	
Rssobs	P‐value	Intercept	P‐value	Q statistic	P‐value	Q statistic	P‐value
4.730	0.892	-0.020	0.430	3.051	0.880	3.751	0.879

## 4 Discussion

Epidemiological researches have determined that ED is highly prevalent in men and escalates with advancing age (2). Currently, ED has emerged as a noteworthy health issue, which imposes considerable psychological and financial burdens on patients, potentially leading to anxiety and depression (4). Thus, it is meaningful to identify the risk factors associated with ED and assess individuals who may require proactive preventive measures or early interventions.

With its numerous indications, aspirin is among the most frequently utilized medications globally, and its use is commonplace among males of middle age and beyond (6). The impact of aspirin on ED has held longstanding scholarly interest. Nevertheless, the causal link between aspirin use and ED remains ambiguous. Prior animal studies have faced challenges due to other factors and financial constraints. Conventional epidemiological studies frequently encounter limitations in establishing causality due to confounding elements. Thus, these leaded to the failure in elucidating the causal connection. Hence, this study aimed to explore the causal association between aspirin use and ED by employing a two-sample MR study based on GWAS data.

In this research, we utilized a two-sample MR method to evaluate the potential causal connection between aspirin use and ED. We chose 9 SNPs significantly linked with aspirin use as IVs. Given that IVW and weighted median method hold an edge in preserving superior estimation precision over the MR-Egger method in MR analysis, the outcomes from the MR analysis provided evidence supporting a potential causal association between aspirin usage and ED. The findings suggested that the usage of aspirin could potentially increase the risk of ED. Furthermore, the sensitivity analysis demonstrated the general robustness of these results.

Our findings were consistent with several previous studies. Darshan et al. evaluated the association between non-steroidal anti-inflammatory drug (NSAID) usage and ED risk using prospective data from the placebo branch of the Prostate Cancer Prevention Trial (PCPT). Their findings did not indicate that NSAID use was linked to a lowered incidence of ED. Contrarily, both non-aspirin NSAID and aspirin usage were tied to a statistically significant 16% heightened risk for mild/moderate ED and severe ED respectively (6). According to the results of Boston Area Community Health (BACH) Survey, taking medication containing aspirin was associated with an increased risk of ED in unadjusted analyses (12). Joseph et al. carried out a prospective cohort study involving 80,966 men to investigate the connection between regular NSAID use and ED. The data demonstrated a link between regular NSAID use and ED, aligning with a Finnish cohort study that showed an elevated incidence of ED associated with NSAID consumption (7, 11). Meanwhile, a study revealed that indomethacin, and to a lesser degree diclofenac, could have a negative impact on erectile responses in rats (36).

However, there were some research findings of the relationship between aspirin use and ED were contrary to our results. A prospective randomized double-blind placebo-controlled study involving 184 patients diagnosed with vasculogenic ED(VED) was carried out by Zeki Bayraktar and Selami Albayrak to examine the efficacy of aspirin therapy in individuals suffering from VED. The research implies that aspirin could be a novel therapeutic approach for VED patients, particularly those with elevated mean platelet volume values (13). Evidence from a randomized, double-blind, placebo-controlled study involving 32 men diagnosed with stable bipolar affective disorder (BAD) undergoing lithium maintenance therapy, suggests that aspirin can significantly ameliorate sexual dysfunction associated with lithium in men with stable BAD (37). The attainment and sustenance of an erection are governed by several molecules released during sexual arousal, including nitric oxide (NO) which originates from nerves in the corpora cavernosa (38) and diffuses into the smooth muscle cells within the cavernosa, causing a rise in the levels of cyclic guanosine monophosphate (cGMP) (39). The elevated cGMP concentrations subsequently facilitate the relaxation of the musculature by reducing calcium ion levels within the smooth muscle cells (40). This entire mechanism enhances blood flow and traps a greater volume blood within the penile tissues, thereby strengthening the hydrostatic skeleton of the penis to achieve an erection (41). Aspirin not only suppresses platelet aggregation activity but also directly enhances the functionality of endothelial NO synthase, leading to increased production of NO and subsequent smooth muscle relaxation, thereby enhancing vascular blood flow (10). It was reported that aspirin therapy had the potential to improve ED in rats with diabetes, potentially influencing the mRNA involved in NO protein synthesis, released from nonadrenergic and noncholinergic cavernosal nerves in diabetes (9).

Then, there were also several studies indicating that an irrelevant relationship between aspirin and ED. The earlier BACH survey demonstrated a correlation between medications containing aspirin and an elevated risk of ED in unadjusted analyses. However, in the multivariable analyses, the OR diminished to an insignificant 1.15 (95% CI: 0.72–1.85) (12). A subset study of the ONTARGET/TRANSCEND trials assessed the link between ED and current treatment in patients at high risk of CVD. Consequently, it was found that neither aspirin nor clopidogrel, taken by 89.2% of the patients, increased the risk of ED (42). Concurrently, the Qatar trial involving male stroke patients showed that nearly 48.3% reported some level of ED. Aspirin was the most frequently used medication among the survivors, with similar usage rates among patients with (75%) and without ED (78%) (43). Moreover, a study using adult rats and aging rat models revealed that long-term aspirin administration did not affect ED (44).

Prostaglandins (PGs) are critical in regulating penile erection (37). Their synthesis consists of two stages. Initially, arachidonic acid (AA), freed from cell membranes, is transformed into an unstable endoperoxide (PGG2) by PG endoperoxide synthase or COX (8). This step is succeeded by the conversion of peroxidase to peroxide and then its transformation to endoperoxide (PGH2). Rapidly isomerized unstable PGH2 results in biologically active end-products such as PGI2, PGE2, prostaglandin D2 (PGD2), prostaglandin F2α (PGF2α), and thromboxane A2 (TXA2) via the corresponding synthase (8, 45). PGs showcase extensive effects and regulate nearly all biological functions. As an illustration, PGI2, the primary AA metabolite in vascular walls, is a highly potent relaxant agent in human penile arteries (46). During the penile erectile process, PGI2 and PGE1 bind to the endothelial PGI receptor (IP) and PGE receptor (EP, mainly EP2/EP4), respectively, activating the Gs protein-coupled receptor (36, 45–47). This activation triggers adenylyl cyclase, which escalates intracellular cyclic adenosine monophosphate (cAMP) levels. The increased cAMP consequently stimulates protein kinase A (PKA) to initiate a phosphorylation cascade, inhibiting myosin light chain kinase, resulting in trabecular smooth muscle relaxation and vasodilation (45, 48). PGI2, due to this relaxing ability, is considered a viable candidate for ED treatment, while repeated intracavernous or intraurethral injections of PGE1 can enhance neuronal and endothelial NOS proteins to improve ED (45).The vasorelaxation properties of PGI2and PGE1 aid in improving ED. Since aspirin inhibits the COX pathway which reduces these vasorelaxation PGs and disrupts NO biosynthesis, it can be logically considered as a risk factor for ED (8, 49).

This study boasted several merits. Firstly, the use of MR analysis, a genetic epidemiological technique, employed instrumental variables for investigating the causality of exposure (use of aspirin) on the outcome (ED). Secondly, the MR principle guaranteed the absence of bias induced by confounders and forestalled reverse causality in the MR analysis. The incorporation of published GWAS data also ensured a substantial sample size and offered significant genetic variation information. As a result, the findings of this study indicated a potential causal association between the use of aspirin and ED, suggesting that aspirin consumption might elevate the risk of ED. And the results were generally robust based on the sensitivity analysis. Lastly, our study participants were drawn from the European population, thereby mitigating potential bias due to population structure.

However, our research had certain constraints. Firstly, the GWAS summary data included only individuals of European descent, hence our conclusions may be primarily applicable to European cohorts. Consequently, exercising caution becomed necessary when applying our findings to racially and ethnically diverse groups. Secondly, this study did not distinguish between the different subtypes of ED (non-vascular or vascular). Future research could focus on analyzing ED within these distinct subgroups. Thirdly, due to data resource restrictions, the conduction of analyses stratified or adjusted for additional covariates was not feasible. Future prospective randomized controlled trials could offer more comprehensive insights and validate the conclusions drawn in this research.

## 5 Conclusion

In our research, we provided evidence that supports a potential genetically determined causal association between aspirin use and the risk of ED among the European population, employing two-sample MR analysis. Our findings suggested that the use of aspirin was associated with an increased risk of ED. Further mechanistic investigations are required to elucidate the intricate connection between aspirin usage and escalated ED risk.

## Data availability statement

The original contributions presented in the study are included in the article/**Supplementary Material**. Further inquiries can be directed to the corresponding authors.

## Author contributions

RL: Conceptualization, Data curation, Formal analysis, Investigation, Software, Writing – original draft. LP: Conceptualization, Data curation, Formal analysis, Investigation, Software, Writing – original draft. DD: Formal analysis, Software, Writing – original draft. GL: Funding acquisition, Methodology, Project administration, Resources, Supervision, Writing – review & editing. SW: Funding acquisition, Methodology, Project administration, Resources, Supervision, Writing – review & editing.
