# Supplementary material for: Potential causal association between aspirin use and erectile dysfunction in European population: a Mendelian randomization study
Source: Front Endocrinol (Lausanne). 2024 Jan 8;14:1329847. doi: 10.3389/fendo.2023.1329847 (PMC10800513; doi:10.3389/fendo.2023.1329847)
Supplement: Supplementary file 1 [file DataSheet_1.docx]

Supplementary Material

# Supplementary Table

**Supplementary Table 1** Details of instrumental variables utilized in the MR analysis of aspirin use on erectile dysfunction.

| SNP | Chr | Pos | Effect allele | Other allele | Exposure (Aspirin use) | | | | Outcome (Erectile dysfunction) | | |
| --- | --- | --- | --- | --- | --- | --- | --- | --- | --- | --- | --- |
|  |  |  |  |  | Beta | SE | *P*-value | *F*-statistics | Beta | SE | *P*-value |
| rs10455872 | 6 | 161010118 | G | A | 0.014522 | 0.001547 | 6.31E-21 | 88.08295 | 0.068 | 0.0375 | 0.07018 |
| rs1171557 | 1 | 156451264 | T | C | 0.005689 | 0.001009 | 1.74E-08 | 31.769275 | 0.0365 | 0.0233 | 0.1165 |
| rs117733303 | 6 | 160922870 | G | A | 0.019747 | 0.003118 | 2.41E-10 | 40.101593 | 0.0629 | 0.0703 | 0.3709 |
| rs1831733 | 9 | 22076071 | C | T | 0.007602 | 0.000849 | 3.41E-19 | 80.191771 | 0.0094 | 0.0218 | 0.6652 |
| rs4694912 | 4 | 49082735 | G | C | -0.0051 | 0.00093 | 4.18E-08 | 30.066496 | 0.0085 | 0.0214 | 0.6912 |
| rs583104 | 1 | 109821307 | T | G | 0.006281 | 0.001008 | 4.64E-10 | 38.825566 | 0.007 | 0.0231 | 0.763199 |
| rs6707197 | 2 | 158900780 | C | A | -0.00519 | 0.000942 | 3.57E-08 | 30.370578 | -0.0119 | 0.0218 | 0.5852 |
| rs73015016 | 19 | 11191300 | A | G | -0.00765 | 0.001304 | 4.50E-09 | 34.394584 | -0.0238 | 0.0307 | 0.4391 |
| rs7412 | 19 | 45412079 | T | C | -0.00865 | 0.001549 | 2.30E-08 | 31.22291 | -0.0557 | 0.035 | 0.1122 |

# Supplementary Figures


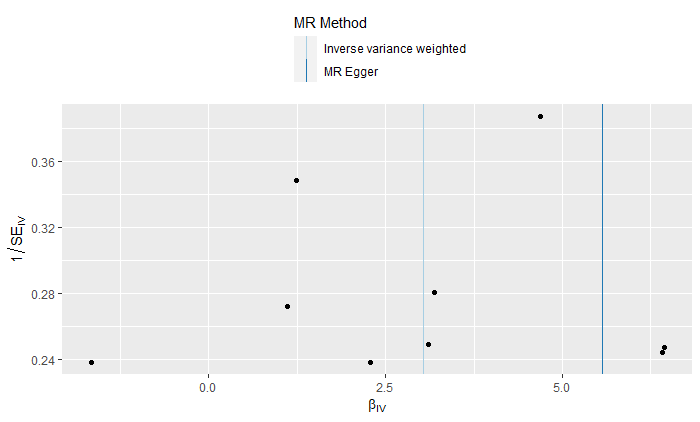


**Supplementary Figure 1** Funnel plot for instrumental variables to assess heterogeneity.
